# Severe Nonketotic Hyperglycinemia in Twins Caused by *GLDC* Variants: The Importance of Accurate Prenatal Variant Interpretation, Counseling, and VUS Disclosure

**DOI:** 10.1002/pd.70186

**Published:** 2026-05-26

**Authors:** Christopher Connolly, Brian Muchmore, Mallory Wagner, Zineb Ammous, Mohannad Ibrahim, Catherine E. Keegan, Elizabeth G. Ames

**Affiliations:** ^1^ Division of Pediatric Genetics, Metabolism, and Genomic Medicine Department of Pediatrics University of Michigan Health System Ann Arbor Michigan USA; ^2^ The Community Health Clinic Shipshewana Indiana USA; ^3^ Department of Radiology University of Michigan Health System Ann Arbor Michigan USA

**Keywords:** carrier screening, GLDC, nonketotic hyperglycinemia, reproductive decision‐making, variant interpretation, variant of uncertain significance

## Abstract

- What is already known about this topic?
  ◦ Nonketotic Hyperglycinemia is a severe metabolic disorder caused by disease‐causing variants in *GLDC* or *AMT*
  ◦ Carrier screening does not universally report variants of uncertain significance (VUS)
  ◦ Genetic counseling is essential for informed reproductive decision‐making
- What does this study add?
  ◦ Highlights the impact of unreported VUS in carrier screening, and the need for accurate variant interpretation and counseling
  ◦ Suggests disclosing a partner's VUS to enhance informed decision‐making when another partner carries a pathogenic or likely pathogenic variant for the same condition

## 1 Fetal Phenotype

A 38‐year‐old G5P4 woman with a dichorionic diamniotic twin male pregnancy underwent cesarean section at 34 weeks of gestation following preterm premature rupture of membranes (72 h) and preterm labor. Antenatal steroids were administered prior to delivery. At delivery, “Twin A” had talipes equinovarus (HP:0001762), and both infants had hypotonia (HP:0001290), apnea (HP:0002104), and bilateral lower extremity clonus (HP:0002169). They developed respiratory failure (HP:0002878) and required intubation and ventilation. EEG demonstrated seizures (HP:0001250) (Summary in Table 1A).

**TABLE 1A:** Clinical data.

Case	Parental details			Gestation at diagnosis	Phenotypes (HPO terms)	Obstetric history	Family history	Outcome
1	Maternal	Age	38	34 weeks	Hypotonia (HP:0001290) Apnea (HP:0002104) Clonus (HP:0002169) Respiratory failure (HP:0002878) Seizure (HP:0001250) Talipes equinovarus (twin A HP:0001762)	G5P4004 with most recent pregnancy conceived via IVF and complicated by fetal hiccups	Unremarkable	C‐section delivery of fraternal twin males with hypotonia (HP:0001290), apnea (HP:0002104), Clonus (HP:0002169), Respiratory failure (HP:0002878), Seizure (HP:0001250), and talipes equinovarus (twin A HP:0001762)
		Ethnicity	Caucasian					
	Paternal	Age	29					
		Ethnicity	Caucasian					

Prenatally, the parents pursued 502‐gene carrier screen panels through a commercial genetic testing company that offered both clinician‐ordered and direct to consumer carrier testing. The father was identified to be a carrier of Nonketotic Hyperglycinemia (NKH, HP:0008288; *GLDC* c.1545G > C, p.(Arg515Ser) Pathogenic, NM_000170.3), factor VII deficiency, and infantile cerebral and cerebellar atrophy. The mother was not identified to be a carrier for NKH but was found to be a carrier of biotinidase deficiency, Cohen syndrome, congenital adrenal hyperplasia, and an intermediate allele for fragile X syndrome. The parents believed there was no risk of affected offspring as they carried different conditions. Prenatal labs and ultrasounds were reportedly normal, but pregnancy was complicated by “fetal hiccups,” a known indicator of NKH.

## 2 Diagnostic Method

A workup evaluated infectious, metabolic, and neurological causes. Rapid trio genome sequencing (WGS) was completed for the children through a clinical diagnostic laboratory. DNA was sequenced using paired‐end reads on an Illumina platform (average 65× coverage). Data were aligned to GRCh38 and analyzed for sequence and copy number variants. Significant variants were confirmed via orthogonal methods (Sanger sequencing). Variants were reported per the American College of Medical Genetics (ACMG) guidelines as described in Table 1B [1].

**TABLE 1B:** Trio whole genome sequencing results.

Procedure (gest Age)	Direct/culture?	Performed test	Secondary confirmatory test	Gene (name; REFSEQ)	Known disease (OMIM)	Variant	ACMG classify‐cation	Criteria applied [2]	Inheritance & zygosity	Interpretation
Serum	Direct	Trio whole genome sequencing	Sanger sequencing	GLDC (NM_000170)	Nonketotic hyperglycinemia (# 238300)	Allele 1: c.1545G > C, p.(Arg515Ser)	Pathogenic	PM2_Moderate PM3_Moderate PP3_Moderate PP4_Supporting PP5_Very_Strong	Autosomal recessive; heterozygous	Pathogenic
						Allele 2: c. 2186C > A, p.(Ala729Glu)	Likely pathogenic	PM1_Moderate PM2_Moderate PP3_Moderate	Autosomal recessive; heterozygous	Likely pathogenic

## 3 Diagnostic Results

Plasma and cerebrospinal fluid (CSF) amino acid analyses revealed elevated glycine levels. Twin A demonstrated plasma glycine of 1876 μmol/L and CSF glycine of 287 μmol/L (CSF/plasma ratio 0.15). Twin B demonstrated plasma glycine of 1898 μmol/L and CSF glycine of 261 μmol/L (CSF/plasma ratio 0.14). Ratios were consistent with NKH (severe NKH 0.09–0.45, attenuated NKH 0.04–0.22), compared to normal ≤ 0.02 [3]. MRIs were consistent with NKH Figure 1A–D.

**FIGURE 1:**
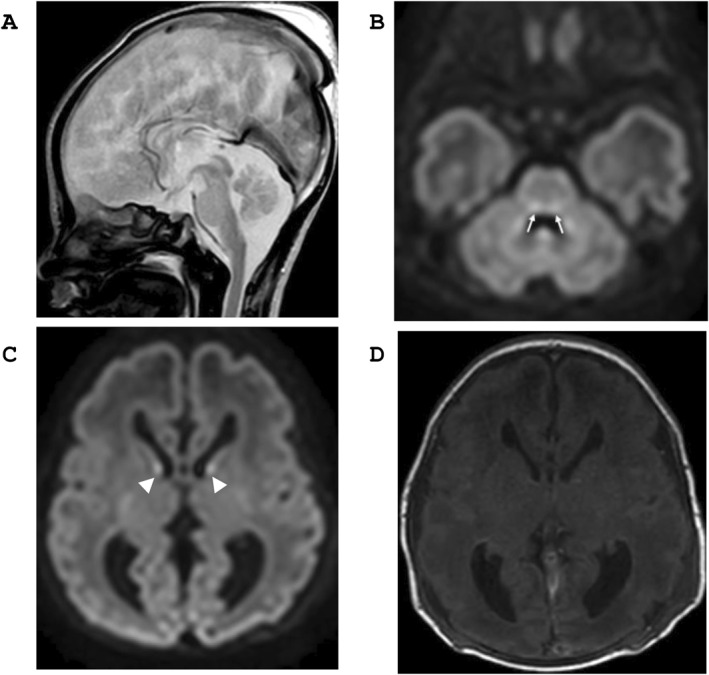
Postnatal brain MRI of twin A. (A) Sagittal T2‐weighted‐image demonstrated abnormal T2‐hyperintensity in the dorsal brainstem. Axial DWI‐image in the pons (B) and the basal ganglia level (C) demonstrated DWI hyperintensity involving the tegmental tracts (arrows) and the expected germinal matrix (arrowheads). (D) Axial T1‐weighted MR image shows loss of the expected T1 hyperintensity in the posterior limbs of the internal capsules, suggesting abnormal myelin.

WGS revealed a known paternal pathogenic variant in *GLDC* (c.1545G > C, p.(Arg515Ser)), and an unknown likely pathogenic variant inherited from the mother (*GLDC* c.2186C > A, p.(Ala729Glu), NM_000170.3; see Table 1B for full variant details) in both infants. The maternal variant was interpreted as likely pathogenic under ACMG variant classification guidelines independent of phenotype [2]. It was determined that the mother's variant was inappropriately classified as a variant of uncertain significance (VUS) on prenatal testing. The company ceased operations in the interval between parental testing and the twins' birth, precluding explanation of variant interpretation. The clinical picture was suggestive of severe NKH, an inborn error of glycine metabolism with deficient activity of the glycine cleavage enzyme system.

## 4 Pregnancy Outcomes and Neonatal Findings

Medical staff disclosed that the twins' presentation was concerning for severe NKH and that treatment is ineffective in altering the progression of profound developmental delays and epilepsy [3]. The family decided to withdraw care, and both infants died on days of life 19 (twin A) and 20 (twin B).

## 5 Discussion

This report emphasizes the importance of accurate variant interpretation and thorough counseling when one parent carries a pathogenic variant for a severe condition. VUS is not universally reported in carrier testing. While over‐reporting and parental anxiety are concerns, given the devastating effects of conditions tested on carrier screening, and when one parent has a pathogenic variant, the disclosure of a partner's corresponding VUS and indication of potential pathogenicity may promote informed decision‐making. Commercial carrier screening results should also be reviewed by a clinical geneticist or genetic counselor before reproductive planning.

## Funding

The authors have nothing to report.

## Ethics Statement

The authors have nothing to report.

## Consent

Written parental consent was obtained to share the details of this case.

## Conflicts of Interest

The authors declare no conflicts of interest.

## Data Availability

The data that support the findings of this study are available on request from the corresponding author. The data are not publicly available due to privacy or ethical restrictions.
